# Fish Collagen Hydrolysates in Wound Healing: A Patent Review

**DOI:** 10.1021/acsptsci.6c00186

**Published:** 2026-07-20

**Authors:** Pamela Cunha Bortoluzzi, Rebeca Mello Chaves, Cecília de Azevedo Marques, Maíra Barroso Pereira, Lana Grasiela Alves Marques, Rodrigo Silveira Vieira, Fábia Karine Andrade

**Affiliations:** † Department of Chemical Engineering, Federal University of Ceará, Fortaleza, Ceará 60455-760, Brazil; ‡ Department of Biochemistry and Molecular Biology, Federal University of Ceará, Fortaleza, Ceará 60455-760, Brazil; § Postgraduate Program in Translational Medicine, Department of Medicine, Paulista School of Medicine, Federal University of São Paulo, São Paulo, Ceará 04023-062, Brazil; ∥ Secretariat for Science, Technology and Higher Education. Secretariat for Science, Technology and Higher Education, Fortaleza, Ceará 60811-520, Brazil; ⊥ Postgraduate Program in Natural Resources Biotechnology, Federal University of Ceará, Fortaleza, Ceará 60440-970, Brazil

**Keywords:** collagen, fish collagen, peptides, wound healing

## Abstract

The wound healing process typically consists of four stages; however, it can be delayed by several external factors, including bacterial contamination, irritation from dressings, and internal conditions such as diabetes, etc. These challenges have driven a growing interest in advanced dressings incorporating bioactive materials, which can protect the wound while simultaneously promoting healing. Collagen peptides derived from collagen hydrolysis have gained increasing attention in this context due to their bioactive properties. Notably, fish collagen has gained prominence in recent years due to its favorable profile compared to traditional sources. These attributes make fish collagen peptides highly advantageous for use in wound care. In this context, literature and patent searches were conducted to assess the application of these materials in the context of biomaterial production and wound healing. An initial screening resulted in 289 patents from the European Patent Office and Cortellis Drug Discovery Intelligence, of which 10 were deemed eligible. The largest concentration of patents originated in China (*n* = 6), Japan and Taiwan (*n* = 2 for both). These patents focused on topical cosmetics and oral formats, which are cosmetic products without a primary focus on therapeutic use. The effects cited in the patents include cell and collagen proliferation, skin hydration, scar repair, antiaging, antioxidant activity, etc. Therefore, it was concluded that there has been an increase in the application of collagen peptides in recent years and that there are several benefits regarding the appearance and repair of the skin, making it interesting to conduct in-depth future research, including clinical trials.

## Graphical abstract

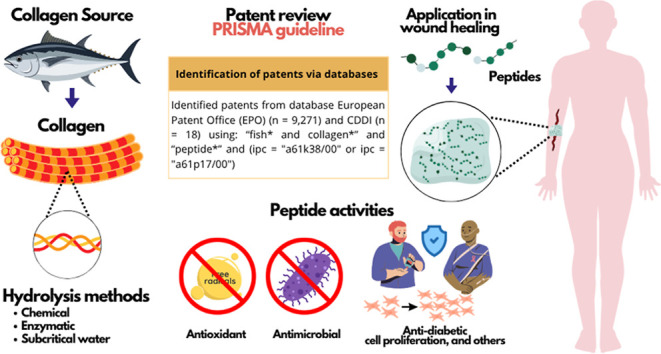

1

The skin, as the largest organ of the human body, acts as a primary protective barrier against mechanical, chemical, and microbial insults. It also plays essential roles in thermoregulation, maintaining hydration, and immunological defense.^1^ Damage to this barrier compromises these functions and may lead to severe local and systemic consequences.

Wounds are defined as disruptions in the standard anatomical structure and function of the skin, often caused by trauma, surgery, burns, or associated diseases.^2^ Its healing is a complex and dynamic biological process involving four sequential yet overlapping phases: hemostasis, inflammation, proliferation, and remodeling. During hemostasis, vascular constriction and clot formation occur to prevent blood loss. The inflammatory phase is characterized by platelet activation and the recruitment of immune cells to eliminate pathogens and cellular debris. Proliferation involves the activation of fibroblasts, angiogenesis, and the formation of granulation tissue, while the final phase, remodeling, can extend for months to years, ultimately restoring tissue strength and function. In some cases, excessive collagen deposition during this phase may lead to hypertrophic scarring or keloids.^200,201^

The duration of the healing process determines whether the wound is categorized as acute or chronic. Acute wounds typically proceed through a timely and organized process of tissue repair. In contrast, chronic wounds exhibit a disruption or delay in this process, failing to restore the skin integrity within an expected time frame—typically within three months.^3^ This condition is particularly prevalent among individuals with certain predispositions, such as the elderly, those with immunosuppression, vascular problems and diabetic patients, representing a significant public health concern.^4^

Globally, it is estimated that over 2% of the population in developed countries will experience a chronic wound at some point during their lifetime.^5^ One study found that the number of US Medicare beneficiaries with wounds increased from 8.2 million to 10.5 million over 5 years (2014–2019). Wound prevalence increased by 13% and expenditures decreased from $29.7 billion to $22.5 billion. Except for venous ulcers, where costs per beneficiary increased from $1206 to $1803, costs per wound decreased, with surgical wounds remaining the most expensive to treat (2014: $3566; 2019: $2504), and the most considerable reduction for arterial ulcers ($9651 to $1322).^6^ These costs include long-term medical care, wound management, hospitalizations, and indirect economic burdens such as productivity loss and decreased quality of life. The increase in wound prevalence alongside the reduction in overall expenditures may be related to coding changes (e.g., ICD-10), the reallocation of care from high-cost hospital outpatient departments to lower-cost physician offices, and reduced use of expensive advanced therapies, particularly for arterial ulcers, which showed the largest cost decrease.^6^

To promote wound healing and reduce complications, dressings are applied to protect the wound from external contaminants, maintain a moist environment, and support cellular activities. They may adhere to the wound bed, cause mechanical trauma during removal, and are generally ineffective at maintaining adequate hydration.^7^ In contrast, advanced dressings—particularly those incorporating bioactive biopolymers—provide significant benefits by addressing these limitations and actively contributing to the healing process.^8^

Among the most promising biopolymers used in modern wound dressings are alginate, chitosan, cellulose, and collagen. Collagen, in particular, plays a fundamental role in skin architecture and tissue regeneration. Hydrolyzed collagen, or collagen peptides, which can be derived from chemical processes, enzymatic degradation, or even water in a subcritical state, exhibits enhanced bioavailability, antioxidant activity, and immunomodulatory effects.^9^ These biopeptides have demonstrated the ability to stimulate fibroblast proliferation, enhance neovascularization, and promote extracellular matrix remodeling, accelerating wound closure and improving cosmetic outcomes.^10^

Marine sources, particularly fish, have gained increasing attention as sustainable and efficient alternatives for collagen extraction. Fish skin, scales, and bones—often considered industrial waste—contain high concentrations of type I collagen. Collagen derived from fish is biocompatible, less immunogenic, and suitable for use in various medical applications, including wound healing.^11^ In addition to environmental and ethical advantages, fish collagen extraction contributes to the valorization of marine byproducts and aligns with the principles of a circular bioeconomy.^12^

Despite the growing body of research highlighting the therapeutic potential of fish collagen hydrolysates in wound healing, there is limited systematic mapping of the corresponding intellectual property landscape. Patents represent a critical link between innovation and practical application, offering insights into the technological maturation of a field, regional innovation trends, and the commercial readiness of specific products. Understanding the global panorama of patents related to fish collagen hydrolysates in wound care is essential for researchers, clinicians, and policymakers seeking to foster innovation, guide investments, and expand access to advanced wound treatments.

Therefore, this review seeks to permeate the current scenario of collagen sources and their hydrolysis methods, as well as the activities found in these hydrolysates, in addition to identifying and analyzing existing patents related to fish collagen hydrolysates for wound healing worldwide. By mapping the geographical distribution, technological scope, and key applicants involved, this study provides a comprehensive overview of the current state of innovation in this field, while also highlighting gaps and potential opportunities for future research and development.

## 2 Fish Collagen Hydrolysates

### 2.1 Collagen Sources

Collagen is a natural, versatile, and invaluable protein in the human body, constituting approximately 25% of total body protein. It plays a fundamental role in various tissues, including cartilage, bones, blood vessels, skin and other connective tissues. Notably, collagen accounts for about three-quarters of the dry weight of skin, over 90% of tendon and corneal tissues and nearly 80% of the organic matter in bones.^13,14^

This essential protein is classified into 29 subtypes, with type I, II and III being the most abundant, comprising 80–90% of all collagen types.^14^ These subtypes are widely distributed throughout the human body and have multifaceted functions, participating in the growth, repair, and maintenance of tissues while also contributing to tissue architecture and resistance.^15–17^

Among these, type I collagen remains the most abundant, accounting for over 70% of the entire collagen family and more than 90% of the collagen found in the human body. Type I collagen is a hierarchically organized protein and is very present in the dermal extracellular matrix.^15,18,19^ Additionally, type I collagen is among the primary polymers used to produce wound dressing hydrogels.^20^

Collagen is a complex protein with a distinct structure that contributes to its unique properties. It consists of three polypeptide chains known as α-chains, organized in a triple-helical conformation featuring characteristic amino acid sequences. These chains form a unit with a molecular weight of approximately 300 kDa, a diameter of around 14–15 Å, and a length of roughly 2800 Å. This unique structure and organization are fundamental to collagen’s role in providing structural support, strength, and flexibility to various tissues in the human body.^15,21^

The self-organization of collagen into fibrils within the extracellular matrix (ECM) ensures the mechanical elasticity of tissues and provides crucial support for cellular activities.^10,22^ Additionally, its intrinsic bioactivity, biocompatibility and biodegradability make collagen a highly desirable biomaterial in tissue engineering.^15^

The primary sources of collagen include swine, bovine, chicken, and fish; however, the first three are the most traditional. For instance, bovine source presents well-established advantages, including biocompatibility and the fact that they do not usually trigger an immune response in most people.^23^

Many studies have focused on extracting collagen from the bones and skin of various bovine animals, including cows, oxen, buffalo and cattle. An interesting aspect of these bovine sources is that some studies have shown their hydrolysates to possess antimicrobial, antioxidant, and antihypertensive activities.^24,25^

Porcine collagen is known for its extremely low immunogenicity, as well as its safety and biocompatibility. When extracted from the skin, it presents economic advantages due to its ease of obtaining.^26^ Porcine collagen is commonly used in dental products that facilitate bone reconstruction and preservation and is found in commercially available membranes such as Jason® and Bio-Gide®.^27^

Additionally, chicken is a valuable source of collagen, particularly from parts such as feathers and feet. Chicken collagen exhibits superior structural properties, including thermal stability and higher yield, compared to bovine collagen. This source also faces fewer religious restrictions than pork collagen.^23^ Furthermore, hydrolysates derived from chicken bone collagen have demonstrated antiaging potential, increased skin antioxidant levels, and reducing inflammation.^28^

However, using traditional sources of collagen (such as swine, bovine, and chicken) is subject to sanitary and sociocultural restrictions due to zoonotic diseases that specifically affect these animals. These include bovine spongiform encephalopathy (BSE), transmissible spongiform encephalopathies (TSE), foot-and-mouth disease (FMD), and avian influenza, as well as classical swine fever virus (CSF) associated with swine sources.^11,29–31^ In addition to these factors, religious and cultural restrictions in some communities regarding the consumption of products derived from traditional sources can further limit the use of collagen. For example, in Islam, Hinduism, and Judaism, the use of materials from bovine, porcine, and poultry origins is forbidden.^32^

As a result, collagen sources of aquatic origin have been gaining prominence in recent years as an alternative to traditional sources. These aquatic sources offer several advantages, including a more straightforward extraction process, a reduced risk of disease transmission, and abundant availability, as they are derived from parts that are typically discarded, such as bones, scales, and skin.^11,29,30,33^

Another favorable factor for obtaining collagen from aquatic sources is the increasing global population and consumption, which has led to a rise in fishing and aquaculture activities, resulting in a substantial amount of fish waste (including skin, scales, and bones) produced each year.^10^ Waste from the fishing industry is becoming a prime target for collagen extraction, particularly from skin, which is often considered low-value material. Furthermore, this fish waste contributes to pollution and has become a public concern due to its offensive odor. Thus, utilizing fish waste for collagen extraction is not only beneficial for resource utilization but also for the environment, as it promotes the more effective use of industrial waste and byproducts from the fishing industry.^31,34,35^

The efficiency of collagen extraction methods from animal byproducts varies based on the source of extraction, the condition of the processed byproducts, and the technology employed.

In general, as highlighted in several patents, collagen offers valuable benefits for use in a wide range of products, particularly in cosmetics. In addition to supporting skin repair, it is often associated with skin-lightning and antiaging effects. However, when administered orally, collagen tends to undergo decomposition, which limits its effectiveness. For this reason, there is growing commercial interest in developing topical applications and alternative delivery systems to harness these associated benefits.^36^

### 2.2 Hydrolysis Techniques

One effective method for utilizing collagen is hydrolysis, which breaks it down into low-molecular-weight proteins, typically less than 10 kDa and potentially reaching below 1 kDa. These low-molecular-weight proteins are commonly referred to as collagen peptides. Hydrolysis can be achieved through various methods, including chemical processes using acids and/or bases, enzymatic treatment, or even by applying water in a subcritical state.^37–39^

#### 2.2.1 Chemical Hydrolysis

Chemical hydrolysis can be divided into two types: acidic and alkaline.^40^ Acid hydrolysis can be performed using inorganic acids, such as hydrochloric acid (HCl), as well as organic acids, including acetic acid. Generally, organic acids are most used due to their ability to dissolve collagen cross-links and allow greater solubilization of this protein.^41^ This method of hydrolysis is considered simple, scalable and relatively low-cost. However, it has several limitations, including low yield, low specificity, the formation of environmentally harmful residues, and the potential degradation of specific amino acids that comprise collagen. These drawbacks render acid hydrolysis less favorable than other hydrolysis methods.^24,41^

In a study conducted by Wisuthiphaet, Kongruang, and Chamcheun,^42^ fish collagen was hydrolyzed using HCl and compared to hydrolysis with the enzyme papain and the combination of HCl and papain. The results showed that peptides obtained through acid hydrolysis exhibited higher hydrolysis and moisture content; however, a lower protein concentration was detected compared to the sample hydrolyzed by papain.

The alkaline hydrolysis technique utilizes a strong base, such as potassium or sodium hydroxide, and is conducted at high temperatures. This method is often employed to determine the composition of specific amino acids, such as tryptophan, which is degraded by acid hydrolysis. However, alkaline hydrolysis can also lead to the degradation of certain amino acids, including serine, cysteine, and histidine.^40,43^

In the study by Zhao, Long, and Gu,^44^ collagen hydrolysis was performed using calcium hydroxide and Alcalase, and a combination of both methods. The results showed that the collagen hydrolysate obtained via the alkaline method had a high protein concentration of 83.3%, whereas the combined alkaline–enzymatic technique yielded an even higher concentration of 93.8%. Similarly, Hong et al.^45^ investigated collagen hydrolysis using formic acid and papain, comparing it to hydrolysis performed solely with papain. Their results indicated that formic acid facilitated the cleavage of cross-links within the collagen structure, promoting the generation of smaller peptides and achieving a yield of 43%, which surpassed that obtained through the conventional enzymatic method. Collectively, these studies demonstrate that chemical hydrolysis, when used in conjunction with enzymatic treatment, can significantly enhance peptide production and improve the overall efficiency of collagen hydrolysis.

#### 2.2.2 Hydrolysis with Subcritical Water

Subcritical water hydrolysis is a process that combines high temperature and pressure, offering several advantages over conventional hydrolysis methods, an environmentally friendly (“green”) technique, as it produces minimal waste and degradation byproducts and eliminates the need for toxic solvents typically used in chemical hydrolysis. Additionally, this method enables faster reaction rates and efficient peptide production.^46^

Ahmed and Chun^38^ applied the subcritical water methodology as a green alternative for collagen extraction from tuna skin. In their study, the sample-to-liquid ratio was 1:200 (w/v), with temperatures ranging from 120 to 300 °C and corresponding pressures of 50 bar (below 280 °C), 80 bar (at 280 °C), and 100 bar (at 300 °C), under continuous stirring at 150 rpm. SDS-PAGE analysis revealed that at 120 °C, the hydrolysate exhibited multiple high-molecular-weight bands, whereas at 180 °C, a distinct 10 kDa band appeared. The highest antioxidant and antimicrobial activities were recorded at 280 °C, corresponding to peptides with the lowest molecular weight (∼600 Da). Beyond this temperature, both activities declined.

Asaduzzaman et al.^47^ also utilized subcritical water hydrolysis to extract peptides from the skin and bones of mackerel (*Scomber japonicus*). Using a collagen-to-water ratio of 1:200 (w/v), temperatures between 200 and 250 °C, and pressures from 30 to 70 bar, with agitation at approximately 150 rpm, they obtained hydrolysates with molecular weight ranges of 789.36–1632.92 Da (skin) and 952.30–1368.13 Da (pepsin-solubilized skin collagen). For mackerel bone hydrolysate, the molecular weight ranged from 759.12 to 988.20 Da at 200 °C and 30 bar. Antioxidant assays, including DPPH, ABTS^+^, ferric reducing power, and Fe^2+^ chelating activity, indicated that the hydrolysates displayed significantly higher antioxidant activity compared to the prehydrolyzed samples.

Haq et al.^46^ further explored subcritical water hydrolysis using collagen from Bigeye tuna skin under catalyst-assisted conditions. Different catalysts were tested, including sodium chloride, acetic acid, and sodium bicarbonate, at a concentration of 0.6%. The process was carried out in batch mode, maintaining a solid-to-liquid ratio of 1:200 (w/v), with 150 mL of liquid loaded into the reactor. The mixture was heated to 250 °C and maintained at a pressure of 50 bar using N_2_ gas with continuous stirring at 150 rpm. The results indicated the highest degree of hydrolysis was achieved with sodium bicarbonate, producing peptides with molecular weights ranging from 300 to 425 Da. Correspondingly, this condition also resulted in the greatest antioxidant and antimicrobial activities.

#### 2.2.3 Enzymatic Hydrolysis

Enzymatic hydrolysis employs proteolytic enzymes under relatively mild conditions, making it a widely used and environmentally friendly (“green”) alternative to chemical hydrolysis.^40,48^ The characteristics and bioactivity of the peptides produced through this process depend largely on the enzyme used. Commonly employed enzymes include Alcalase, papain, Neutrase, and Savinase.^49–52^

In the study of Devita et al.,^50^ collagen peptides were extracted from the skin of bigeye tuna (*Thunnus obesus*) using Alcalase, Neutrase and Savinase. Additionally, two groups of collagens were investigated: one solubilized with pepsin and the other with bromelain. The results revealed that pepsin-solubilized fractions exhibited the highest antioxidant activity, while among the enzyme treatments, Savinase produced peptides with molecular weights below 3 kDa and displayed the strongest antioxidant potential.

Furthermore, the enzymatic hydrolysis process can be combined with other methods, as demonstrated in the study by Hao et al.,^53^ where peptides were produced from porcine bone collagen using the ultrasound-assisted enzymatic hydrolysis technique.

One of the advantages of the enzymatic method is the ability to utilize tools, such as BIOPEP, a database that provides information about the most likely bioactive peptides obtainable based on the source and the enzyme used in the hydrolysis. The platform offers peptide sequences and their corresponding activities, with ongoing updates.^54^ An example of this database application is found in the study by Wardani et al.,^49^ which utilized collagen from the skin of yellowfin tuna (*Thunnus albacares*) and conducted in silico tests to identify the most suitable enzyme for in vitro application. The researchers evaluated parameters such as the theoretical degree of hydrolysis (DHt), the frequency of fragments formed with antioxidant activity (*A*_E_), the potential of antioxidant activity in fragments produced (*B*_E_), and the relative activity of fragments exhibiting antioxidant activity (*A*). Based on these assessments, papain was identified as the most effective enzyme for the in vitro study.

### 2.3 Biological Activities

#### 2.3.1 Antioxidant Activity

Antioxidant activity refers to the ability of a natural or synthetic substance to protect against damage caused by free radicals, reactive oxygen or nitrogen species (ROS and RNS), and other unstable molecules. These substances help defend the body against degenerative diseases, cancer, and provide various antiaging benefits.^55–57^

For peptides, several factors can influence their antioxidant activity, including molecular weight, amino acid sequence, and overall composition. Although the mechanisms underlying the bioactive properties of the peptides have not yet been fully elucidated, literature suggests that both hydrophobic and aromatic amino acids, such as histidine and tryptophan, play a major role in the scavenging radicals activity of peptides. Other characteristics, such as the spatial structure of the amino acids present in the sequence and the amphiphilic nature of peptides, can impact on the interaction between the peptide and radical species and are also related to their antioxidant activity.^58,59^

In a study by Zamorano-Apodaca et al.^60^ involving fish byproducts from various species, acid-soluble collagen was hydrolyzed with Alcalase. The analysis revealed that peptide fractions with lower molecular weights (≤1 kDa) exhibited significant antioxidant properties, showing high reducing power with an absorbance of 0.366 and hydroxyl radical scavenging activity of approximately 91% at a concentration of 15 mg/mL. Additionally, at a concentration of 5 mg/mL, these peptides demonstrated excellent DPPH scavenging activity, reaching 81%. The authors associated its activity with the higher amount of donating electron/hydrogen peptides available to react with free radicals in lower weight peptides, and also mentioned the high content of hydrophobic amino acid residues.

Additionally, chemical modifications can be applied to enhance peptide properties, such as phosphorylation. An example is the study by Luo et al.,^61^ which used collagen peptides from fish bones. The DPPH radical scavenging activity was evaluated for four peptide variations: the unmodified peptide (CP), the phosphorylated (P-CP), the calcium-chelated (Ca-CP) and the calcium-chelated phosphorylated (Ca-P-CP). The results showed DPPH scavenging activities of 57.09%, 67.03%, 62.66% and 71.05%, respectively. This demonstrated that modifications, especially in the case of Ca-C-CP, were highly effective in enhancing the hydrogen donating ability of peptides, thus increasing its antioxidant activity.

In addition to studies focused on fish, it is also important to note that antioxidant activity has been observed in collagen peptides from other sources. A study carried out by Mamoudou et al.^62^ analyzed the bioactivity of leather collagen peptides, revealing a high presence of antioxidant activity among the obtained sequences, especially sequences that had very low molecular weight and hydrophobic amino acids in its composition. Another study by Xie et al.,^63^ also involving peptides rich in hydrophobic amino acids from leather collagen, demonstrated several positive results in tests with DPPH, ABTS and hydroxyl radicals, in addition to presenting a high Fe­(III)-reducing power. It is also worth noticing that most of the articles use indirect assays, such as DPPH and ABTS, to evaluate the peptides’ antioxidant activity, and due to limitations of those methods, the activity may not translate into in vivo assays.

#### 2.3.2 Antimicrobial Activity

Antimicrobial peptides (AMPs) have attracted considerable attention for application in wound dressings, particularly due to the rising prevalence of antibiotic-resistant bacteria. As a result, there is an ongoing search for efficient and innovative alternatives for their application. AMPs are typically cationic and exert their antimicrobial activity primarily through disruption of the bacterial cytoplasmic membrane. Beyond their bactericidal properties, AMPs also inhibit biofilm formation, thereby reducing bacterial adhesion and colonization.^64,65^ Moreover, their incorporation into hydrogel-based membranes offers a promising strategy for managing wound infections, as the release kinetics of AMPs can be tuned to ensure sustained antimicrobial activity and prolonged wound protection.^66^

One important consideration is that certain AMPs, such as daptomycin—a 1.62 kDa cyclic lipopeptide—and polymyxin B—a 1.2 kDa cyclic polypeptide—have successfully advanced through preclinical and clinical development stages. Nevertheless, despite the discovery of numerous AMPs, their wider clinical application remains limited. This limitation is primarily attributed to interactions between the peptides and physiological components, such as saline ions and endogenous enzymes, which can reduce their stability and antimicrobial efficacy. Consequently, their use is restricted to specific applications. To overcome these challenges, various modification strategies, such as incorporation into hydrogels or encapsulation within nanoparticles, have been explored to enhance peptide stability, bioavailability, and overall therapeutic performance.^67^ Like other properties, antimicrobial activity is influenced by various factors, including the collagen source, the hydrolysis method used, the amino acid sequence, peptide size, and charge. A key characteristic of antimicrobial collagen peptides is the hydrophobicity of their amino acids, which facilitates penetration into microbial membranes and facilitates their destruction.^68^ Since collagen is a biomolecule rich in amino acids such as proline and glycine,^69^ among other hydrophobic amino acids, the production of antimicrobial peptides with hydrophobic properties is feasible.

In the study conducted by Zamorano-Apodaca et al.,^60^ the antimicrobial activity of the collagen fractions against the *Escherichia coli* was evaluated, where only the fraction ranging from 10 to 30 kDa exhibited inhibitory activity. This fact may be associated with the presence of cationic and hydrophobic amino acids in this fraction, since, normally, peptides with lower molecular weight (<10 kDa) tend to exhibit antimicrobial activity, thus demonstrating that different factors can affect and even increase the antimicrobial capacity of some peptides. Other works associate the antimicrobial activity of collagen hydrolysates with the degree of hydrolysis, since higher hydrolysis degrees can make smaller peptide fractions.^70^ This suggests that collagen peptides have significant potential as effective antimicrobial agents for therapeutic applications.

Furthermore, similar antimicrobial activity has been observed in synthetic peptides, such as Tet213 (1.59 kDa). This synthetic amphiphilic cationic peptide is recognized for its effectiveness against both Gram-positive and Gram-negative bacteria, providing the advantages of stability and potent antimicrobial activity.^65^

#### 2.3.3 Anti-inflammatory Activity

Inflammation is a natural part of the wound healing process; however, in chronic wounds, it can persist for a prolonged period, complicating complete healing.^71^ Chronic inflammation, often seen in conditions like diabetic wounds, atherosclerosis and neurodegenerative diseases, is characterized by the elevated levels of proteins such as inducible nitric oxide synthase (iNOS) and nuclear factor kappa-B (NF-κB).^72^

Numerous studies have reported on the anti-inflammatory effect of peptides. Luo et al.^73^ observed the effect of collagen peptides derived from Atlantic salmon (*Salmo salar*) bones, demonstrating both in vitro and in vivo anti-inflammatory activity. In the in vitro analysis, chondrocytes treated with peptides exhibited a reduction in TNF-α and IL-6 levels of 28.10% and 10.94%, respectively. In the in vivo tests, the results indicated significant reductions in serum levels of IL-6, IL-17, and TNF-α, as well as decreased expressions of TNF-α and IL-1β in cartilage tissue following treatment with collagen peptides.

In other sources of collagen, similar activity has been observed. For instance, Hao et al.^53^ reported significant anti-inflammatory effects in their study on porcine bone collagen peptides. They evaluated the impact of these peptides on the secretion of nitric oxide and inflammatory markers TNF-α and IL-6. The results showed that all three markers exhibited a reduction in secretion, with an inhibition of up to 58.31% at a peptide concentration of 10 μg/mL. Likewise, the other activities mentioned in this paper, the anti-inflammatory activity was associated with hydrophobic amino acids present at the C- and N-terminal. Yang et al.^72^ also associated yak bone collagen peptides with interaction with the NF-κB pathway, which alleviates inflammation.

Synthetic peptides have also demonstrated anti-inflammatory activity. A study by Fathi et al.^74^ investigated a new synthetic peptide, mCHTL (131–140), which has previously shown antimicrobial activity. The findings revealed that this peptide induced the production of TGF-β, while reducing TNF-α levels. This suggests a high potential for wound healing applications, as the peptide not only accelerates the healing process but also provides protection against bacteria such as *Acinetobacter baumannii*.

#### 2.3.4 Cell Proliferation Activity

Cell proliferation and the migration of keratinocytes toward the center of the wound are crucial for wound healing, playing a key role in the re-epithelialization process. During this stage, the wound is filled with granulation tissue, which serves as the foundation for keratinocyte proliferation. This new tissue formation typically occurs within two to 10 days after the wound is created.^75^ Numerous studies have demonstrated the positive effects of peptides in promoting cell proliferation during the healing process.

Lee et al.^76^ demonstrated that fish collagen peptides promote cell proliferation. In their research, the CCK-8 cell proliferation assay was employed to evaluate the effects of peptides on thymic epithelial cells, confirming that these peptides induced cell proliferation through the NF κB signaling pathway.

Similarly, a study by Hwang, Park, and Lee^77^ evaluated the effects of peptides extracted from the collagen of Mozambique tilapia scales (*Oreochromis mossambicus*). In vitro tests with human dermal papilla cells (hDPCs) revealed that these peptides enhanced cell proliferation, resulting in increased hair growth. The most significant effect was observed at a concentration of 62.5 ppm, which resulted in a proliferation rate of 113.98 ± 1.44%.

Similar activities have been observed in other sources of collagen. Wu et al.^78^ investigated whether peptides from pig bone collagen could stimulate cell proliferation. They tested four different peptide variations: the standard peptide (CP), the phosphorylated peptide (PCP), the calcium chelate peptide (CP-Ca) and the phosphorylated calcium chelate peptide (PCP-Ca). Using the MC3T3-E1 cells (mouse preosteoblast), the study found an increase in cell viability of approximately 130% to 150%, indicating positive results for cell proliferation.

In another study, Hou et al.^79^ examined the effects of collagen-derived peptides from the skin of the iris squid (*Sthenoteuthis oualaniensis*) on L-929 cells (fibroblasts). The study highlighted that the increase in cell viability was directly proportional to the length of the peptides. Furthermore, a concentration of 0.07 μM was identified as providing the best results for cell proliferation.

#### 2.3.5 Other Biological Activities

In addition to the previously mentioned bioactivities, recent research has revealed effects, including anticancer properties. Inbaraj, Lai and Chen^80^ investigated two collagen peptides derived from tilapia skin, TSCP1 (482 Da) and TSCP2 (172 Da), and their nanoformulations—nanoemulsion (NE), nanoliposome (NL), and nanogold (NG)—as well as folic acid/chitosan-conjugated versions. Their findings demonstrated that these peptides, particularly the nanopeptides, effectively inhibited the growth of human lung cancer A549 cells while showing lower cytotoxicity toward normal lung fibroblasts MRC-5 cells, indicating selective anticancer potential.

Furthermore, research by Hou and Chen^81^ confirmed antidiabetic activity in collagen peptides derived from waste sturgeon fish skin. They prepared nanoemulsions containing low-molecular-weight collagen peptides, among which the LHN nanoemulsion (low-molecular-weight, high-dose formulation) exhibited the most pronounced effects. In diabetic mice, this formulation achieved approximately 95.5% wound-healing area closure and reduced fasting blood glucose levels by about 46.8%. These findings are particularly relevant, as elevated fasting glucose is a hallmark of diabetes.

Additionally, antihypertensive activity has been observed. In the work of Dong et al.,^82^ a peptide was isolated from tilapia skin treated by steam-explosion and enzymatic hydrolysis—specifically VGLFPSRSF (∼1009.17 Da)—which exhibited a strong inhibitory effect on angiotensin-converting enzyme (ACE, IC_50_ ≈ 61.43 μM) and, based on molecular-docking and network-pharmacology analyses, was shown to interact with hypertension-related targets (e.g., ACE, AKT1, REN), thereby indicating its potential as an antihypertensive agent.

Furthermore, as demonstrated in the study by Huang et al.,^83^ collagen peptides from the skin of silver carp (*Hypophthalmichthys molitrix*) also exhibited antiphotoaging bioactivity. The enzymatic hydrolysate contained peptides with molecular weights ranging from 358.68 to 863.89 Da, including the sequences: GPPGPPGTPGPQ, SGLPGPIGPPGPR, and GLPGPIGPPGPR, which showed protective effects against UVB-induced photoaging. At a concentration of 0.1 mg/mL, the peptide mixture restores the viability of UVB-irradiated L929 fibroblast cells, indicating its potential as an antiphotoaging agent.

## 3 Patent Analysis

Intellectual property plays a strategic role, reflecting both technological maturity and the interest of different economic and institutional actors in markets considered promising.^84^

In this context, patents provide researchers, companies, and governments with valuable insights for planning technological development and building scenarios for emerging technologies, based on the analysis of technical information contained in these documents. The results of this type of analysis generate indicators regarding technical characteristics, market trends, and industrial innovation strategies, which may guide public investment decisions, policy formulation, and even corporate strategies such as mergers and acquisitions.

### 3.1 Data Collection

For the collection of data related to the mapping of technological information, the following databases were used: Espacenet Patent Search from the European Patent Office (EPO), the United States Patent and Trademark Office (USPTO), and the Cortellis Drug Discovery Intelligence (CDDI) Database (Clarivate Analytics). The search strategy was based on keywords using the terms: fish AND collagen AND (hydrolysis* OR peptide*) AND (“wound healing” OR “tissue repair” OR “skin regeneration”)*.

To refine the large number of retrieved documents (EPO (*n* = 9271) and CDDI (*n* = 18)), the search was restricted to the Title/Abstract fields. The survey was conducted in February 2025. After the execution of the search strategy in the databases, a data treatment stage was carried out. During this stage, the PRISMA method (Preferred Reporting Items for Systematic Reviews and Meta-Analyses) was applied.

## 4 Technological Survey of Fish Collagen Hydrolysates in Wound Healing

Figure 1 illustrates the systematic search and screening strategy used in this study, which was based on the PRISMA method, developed to provide minimum reporting items for the organization of systematic reviews.^85^

**Figure 1:**
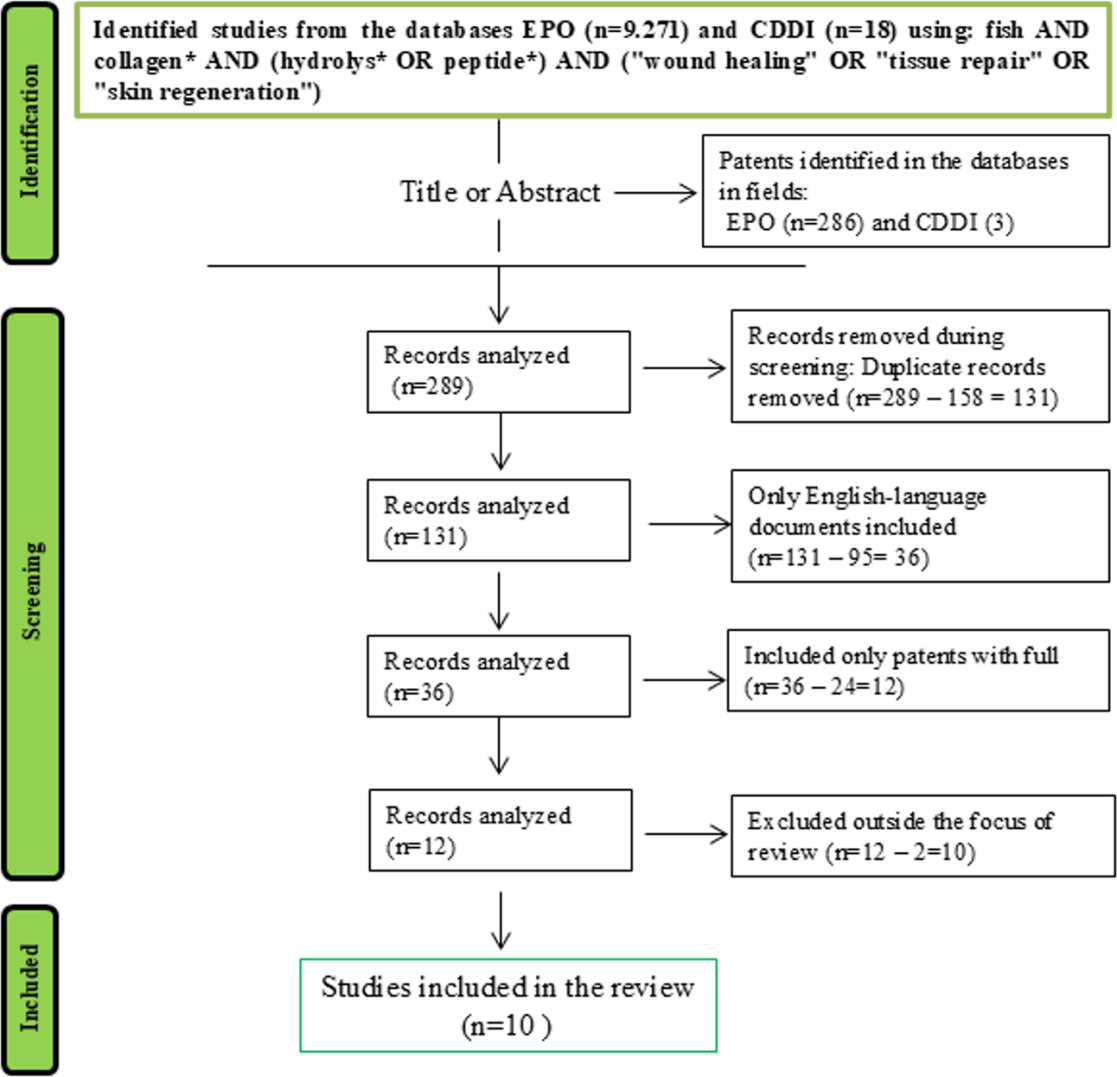
Flowchart of patent search for fish collagen peptides and screening. *n* = number of results obtained. Source: Prepared by the authors, PRISMA flow adapted from Page et al.^85^

The mapping identified 9289 patent documents, which were refined to 289 after title/abstract screening, of which 158 patents were duplicates, 95 patents were excluded for not being written in English, most of them originating from China, South Korea, and Japan, and 24 were excluded because the full text was not available.

In addition, after title and abstract screening, 2 patents were excluded for being outside the scope of the review. Therefore, patents related to food compositions and supplements intended for other purposes were excluded. Ultimately, 10 patents were selected for critical appraisal based on wound-healing criteria and related bioactivities (e.g., collagen proliferation and antioxidant activity) (Table 1). The earliest filings date back to 2007, and the latest to 2023. The geographical concentration of patents is China (*n* = 6), Japan (*n* = 2), and Taiwan (*n* = 2), reflecting established R&D hubs in marine-based biomaterials and cosmetics in Asian countries.^36,86–94^

**Table 1:** Selected Patents Using Fish Collagen Peptides for Skin Wound Healing Applications^a^

patent number	applicant (Country)	year	type of formulation	species/fish	field of application	tests	ref
CN101468200B	Dalian Longhe Ocean Bioengineering Technology Co Ltd. (China)	2007	composed of 10–20 parts of type VI deep-sea fish collagen, 10–20 parts of type VII deep-sea fish collagen, 20–30 parts of deep-sea fish collagen peptides, 20–30 parts of deep-sea fish collagen small peptides (<3 kDa), 10–18 parts of pearl calcium powder and 2–10 parts of ginseng powder (by weight)	cod	the invention provides an aesthetic health-care capsule and its preparation, produced from deep-sea fish collagen. It enhances the health-promoting effects of fish collagen. The compatibility of the raw materials contributes to beauty and health benefits from bones to epidermis	NS	Li et al.^86^
JP5184257B2	Hasebe Yukio (Japan)	2008	the invention relates to eight variations of a beverage formulation, where the base consists of eggshell membrane powder, fish-derived collagen peptide, and N-acetylglucosamine as active ingredients	NS	the invention concerns a beverage containing eggshell membrane powder and fish-derived collagen peptides with N-acetylglucosamine, which prevents skin aging and roughness, improves smoothness, softness, moisture, firmness, and luster, and promotes overall health	in vitro and in vivo	Yukio^92^
JP5449730B2	Kyowa Yakuhin K. K. (Japan)	2008	a menopausal disorder improvement agent and nutritional supplement formulated from fish ovarian skin extract available as a drink, granules, or tablets	salmon	provides an agent for improving menopausal disorders and a nutritional supplement prepared by enzymatically hydrolyzing the ovarian skin (from cod, herring, salmon, etc.), effective for menopausal symptom relief and overall health maintenance	in vitro and in vivo	Shinichi^89^
CN102178636B	South China Sea Institute of Oceanology; Foshan Aai Cosmetic Health Care Product Co. Ltd. (China)	2011	a cosmetic formulation containing 10% marine bioactive substances by mass: 8.3% active peptide from wavy clam, 0.1% collagen peptide from cod skin, 1% polysaccharide from laver, 0.6% shark oil, and 90% cosmetic base	cod or other marine species	a marine biological functional cosmetic design for pore refinement. The invention belongs to the field of cosmetics, utilizing marine bioactive extracts as the main active ingredients	in vivo	Sun et al.^91^
CN107397954A	Lanxi Chenmo Biological Tech Co Ltd. (China)	2017	functional small peptides extracted from deep-sea fish are used as protective agents or absorption promoter for collagen in cosmetics, health products, food, feed or medicine	deep-sea fish	the invention discloses the use of functional small peptides extracted from deep-sea fish species for diverse applications	NS	Jianbo^36^
TWI788003B	TCI Co Ltd. (Taiwan)	2020	the invention describes the use of peptides as bioactive substances for antiaging compositions. An example composition contains peptides comprising six amino acid sequences (SEQ ID NO: 1–6)	*Pangasius bocourti*	relates to the use of biologically active peptides for preparing antiaging compositions	in vitro and in vivo	Yung-Hsiang and Yu-Ling^88^
CN112314952A	Chengdu Sanajon Pharmaceutical Group Sichuan Huamei Pharmaceutical Co Ltd. (China)	2020	a composition with blue-light protection and skin-quality improvement functions, consisting of (by weight): 5–50 parts of fish collagen peptide, 0.01–5 parts of bonito elastin peptide, 0.05–0.1 parts of lutein, 0.01–0.1 parts of zeaxanthin, the peptide ratio is 5–10:1 (fish: bonito)	NS	belongs to the field of bioengineering; the composition provides blue-light resistance and improves overall skin condition	in vivo	Haiyan et al.^90^
TWI784298B	Tci Co Ltd. (Taiwan)	2020	a composition that may be used as food, health food, or a pharmaceutical product, containing oral collagen peptide powder	tilapia	relates to the use of a bioactive peptide for the preparation of compositions that improve skin condition	in vitro and in vivo	Yung-Hsiang and Yu-Ling^93^
CN111234007	Inner Mongolia Yongjian Biotechnology Co., Ltd. (China)	2020	describes the use of collagen active peptides for promoting wound healing in external and/or internal medicines the external formulation is a wound plaster, containing collagen active peptides; the oral formulation may include capsules, tablets, granules, or pills	fresh carp and tilapia	the invention provides collagen active peptides that promote wound healing, addressing the issue of underutilized fish scales and skins from aquatic products	in vivo	Yeguo^94^
CN117286207A	University of South China Agriculture (China)	2023	utilizes dried white jade fish maw as raw material, combining fermentation with complex bacterial enzymatic hydrolysis to obtain a small molecular weight, colorless, odorless peptide with antioxidant and moisturizing effects	white jade fish	relates to the field of food fermentation and bioactive peptide preparation, describing a white jade fish maw peptide that improves skin condition	in vitro and in vivo	Pan et al.^87^

a NS: not specified.

The findings are consistent with recent global indicators, which show a continued rise in patent filings and Asian leadership (notably China, followed by Japan and the Republic of Korea) in R&D-intensive areas, such as bioproducts and cosmetics. This provides a macro-context for the density of documents observed in China/Japan/Taiwan.^84^ Such concentration may reflect regulatory and industrial ecosystems that integrate fisheries/aquaculture chains, enzymatic processing, and the skincare industry, facilitating faster value appropriation through patents.

The predominance of industrial applicants (*n* = 7; 70%) over academia (*n* = 1; 10%) and individuals (*n* = 2; 20%) indicates a market-oriented profile and incremental maturity, while underscoring the need for comparable studies (peptide MW/sequence characterization; standardized preclinical models) to substantiate wound-healing claims - especially within A61K38/00 and A61P17/00, the methodological subclasses used in this study (Figure 2).

**Figure 2:**
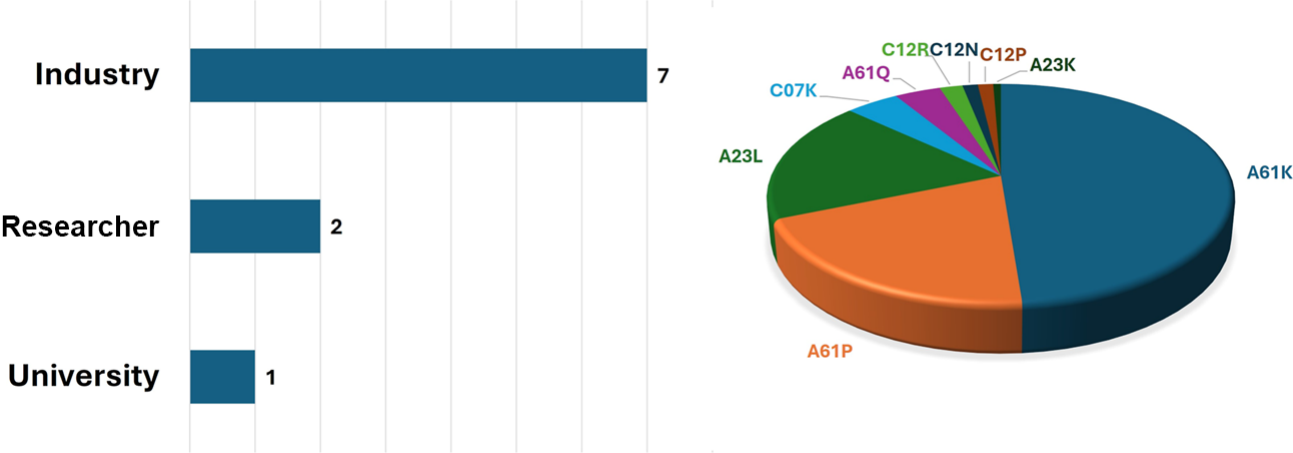
Patent applicant (a) and International Patent Classification (IPC) for the patents selected in the study. Source: Prepared by the authors.

The ten patents can be grouped into three application tracks: (i) topical/cosmetic preparations (A61K/A61Q) targeting pore refinement, hydration, improved firmness, and increased collagen density; (ii) oral nutraceuticals (A23L/A61K)—including capsules or beverages with fish-derived collagen peptides, sometimes combined with N-acetylglucosamine and eggshell-membrane powder; and (iii) peptide/process filings (C07K; C12N/C12P/C12R) focused on raw-material standardization and enzymatic production routes. Within this set, only one patent explicitly targets wound healing with external and oral dosage forms.^94^ Several others reference wound-relevant or adjacent skin-repair effects - for example, claims related to burn healing,^92^ pore refinement,^91^ blue-light protection,^90^ improved moisture/elasticity in menopause,^87–89,91,93^ and general “skin repair” ^36,90,91,94^ - but they are framed primarily as cosmetic or skin-quality outcomes rather than therapeutic wound end points. Overall, the patent compilation evidenced encompasses topical cosmetics and oral formats, as well as combinations (capsules/bioactive matrices for dressings and hydrogels), characterizing a cosmetic–nutraceutical–biomaterial hybridization that occupies a transitional zone between functional skincare and wound care; few claims are grounded in classical histological/functional end points of wound healing.

From this overview, three strategic implications remain salient (i) prioritize functionalized peptide hydrogels (e.g., chitosan/PVA with antimicrobial features): for chronic wounds; (ii) develop combined regimens (topical + oral) with <1 kDa molecular-weight standardization and mechanistic markers (collagenogenesis, angiogenesis, inflammatory modulation) to bridge from cosmetic benefits to wound-specific evidence; and (iii) broaden IP strategies beyond composition claims to include methods of use, combination devices, and “green” production leveraging fishery byproducts—while designing programs that generate wound-specific end points to close the translational gap identified above.

Recent literature supports the biological rationale observed across patents: marine collagen peptides exhibit antioxidant, antimicrobial, and pro-regenerative properties relevant to the phases of wound healing. Notably, <1 kDa fractions tend to demonstrate higher bioavailability and superior performance in preclinical models, including topical and oral applications derived from fish scales. In parallel, peptide-based hydrogels are gaining traction as delivery platforms and pro-healing matrices for chronic wounds, aligning with families that combine peptides with chitosan and PVA.^95^ Regarding nutraceuticals, meta-analyses of RCTs show a modest but consistent benefit of oral collagen peptides for skin hydration and elasticity, which helps explain the number of ingestion-oriented “skin health” filings—although these do not replace condition-specific clinical evidence for wound healing.^96^

This distinction between skin-health claims and wound-healing therapeutics is central to interpreting the translational maturity of the patent landscape identified in this review. Wounds are clinically heterogeneous entities, classified not only as acute or chronic but also according to clinical presentation and underlying etiology, including diabetic foot ulcers, venous leg ulcers, arterial ulcers, and surgical or traumatic wounds, each involving distinct pathophysiological mechanisms and therapeutic needs.^97,98^ This heterogeneity has direct implications for translational development: a bioactive agent that promotes general skin hydration or elasticity does not necessarily address the specific impairments present in a chronic wound environment. Diabetic wounds, for instance, are characterized by persistent inflammation, excessive reactive oxygen species, microbial burden, extracellular matrix degradation, impaired fibroblast and keratinocyte function, reduced angiogenesis, and delayed re-epithelialization, which require targeted mechanistic interventions rather than broad skin-quality effects.^99^ Accordingly, therapeutic claims for wound healing require indication-specific end points, such as wound closure rate, re-epithelialization, granulation tissue formation, collagen deposition and remodeling, angiogenesis, infection control, scar quality, and recurrence, which are not consistently addressed in the patents analyzed.^97,98,100,101^

From a translational perspective, the biological activities attributed to fish collagen hydrolysates, including antioxidant, antimicrobial, anti-inflammatory, and pro-regenerative effects, acquire clinical relevance only when explicitly linked to defined mechanisms of impaired wound repair in specific wound types. Future development should therefore move beyond broad bioactivity screening and incorporate standardized peptide characterization, including mean molecular weight, sequence profile, and degree of hydrolysis; indication-specific preclinical models with relevance to human wound pathophysiology; wound-specific functional end points assessed through histological and biochemical analyses; and systematic biocompatibility and safety assessment aligned with applicable regulatory frameworks.^97,99,101^ The regulatory classification of products containing fish collagen peptides, whether as cosmetics, nutraceuticals, medical devices, drug–device combinations, or therapeutic agents, determines the type and level of evidence required for development and market authorization. Classification as a wound-care medical device or drug–device combination, for example, may require biocompatibility evaluation according to ISO 10993-1 and clinical or performance evidence proportional to the product’s intended use, mechanism of action, and risk profile, whereas cosmetic classification does not impose equivalent wound-healing efficacy requirements.^102^ This distinction should be considered from early stage development to avoid regulatory misalignment and to ensure that innovation in this field generates clinically relevant evidence.

Despite a well-supported biological rationale, the patent landscape analyzed in this review reveals a translational gap. There is a scarcity of clinical trials specifically designed for acute or chronic wound end points to support patent claims. Peptide characterization across patent filings is heterogeneous and inconsistently reported, which limits comparability across studies and hinders regulatory translation. Preclinical programs aligned with biocompatibility standards and regulatory expectations for wound dressings and devices could reduce uncertainty and accelerate the development readiness of hydrogels and bioactive matrices containing fish collagen peptides. Bridging the gap between the cosmetic-nutraceutical orientation of current patents and the clinical evidence required for wound-healing therapeutics will require coordinated investment in mechanistic studies, standardized preclinical models, and well-designed clinical trials targeting indication-specific outcomes.

### 4.1 Study Limitations and next Steps

The search strategy (EPO), descriptors, and IPC filters may underrepresent patents with less explicit wording. Language and jurisdiction biases, as well as the lack of in-depth family analysis (including legal status, citations, and continuations), also limit inferences about value and freedom to operate. Future studies should: (a) expand to a family level landscape (INPADOC; forward/backward citations); (b) apply text-mining to standardize peptide profiles; (c) integrate wound-specific clinical evidence; and (d) map regulatory barriers by product class (device vs cosmetic vs special-purpose food).

## 5 Conclusions

In conclusion, there is significant potential for the use of fish collagen peptides in the wound-healing process. This is especially pertinent given the growing interest in such materials, particularly in response to increasing concerns about the excessive use of antibiotics and the search for effective alternatives to combat resistant bacteria. Furthermore, these peptides offer several additional benefits promoting faster healing and enhancing the appearance of the skin, which addresses aesthetic concerns for patients. These benefits are confirmed by the fact that there has been a growth in the last two decades in the application of peptides, especially from marine sources, in skin treatments, both for local use and for consumption in tablet form. In addition, their application is expanding to medical, food, and cosmetic products, among others, which demonstrates their broad applicability and supports industrial interests.

## Abbreviations

ICD-10: International classification of diseases, 10th Revision
kDa: kilodalton
iNOS: inducible nitric oxide synthase
NF-κB: nuclear factor kappa-B
Å: Ångström
ECM: extracellular matrix
BSE: bovine spongiform encephalopathy
TSE: transmissible spongiform encephalopathies
FMD: food-and-mouth disease
CSF: classical swine fever
HCl: hydrochloric acid
°C: degree Celsius
Da: dalton
w/v: weight by volume
SDS-PAGE: sodium dodecyl sulfate polyacrylamide gel electrophoresis
DPPH: 2,2-diphenyl-1-picrylhydrazyl
ABTS+: (2,2′-azino-*bis*(3-ethylbenzothiazoline-6-sulfonic acid) radical cátion
rpm: revolutions per minute
N_2_: nitrogen
DHt: theoretical degree of hydrolysis
A_E_: frequency of fragments formed with antioxidant activity
*B*_E_: potential of antioxidant activity in fragments produced
*A*: relative activity of fragments exhibiting antioxidant activity
ROS: reactive oxygen species
RNS: reactive nitrogen species
mg/mL: milligrams per milliliter
CP: unmodified peptide/standard peptide
P-CP: phosphorylated peptide
Ca-CP: calcium-chelated peptide
Ca-P-CP: calcium-chelated phosphorylated peptide
PCP: phosphorylated peptide
CP-Ca: calcium chelate peptide
PCP-Ca: phosphorylated calcium chelate peptide
Fe­(III): ferric ion; AMPs—antimicrobial peptides
TNF-α: tumor necrosis factor alpha
IL-6: interleukin-6
IL-17: interleukin-17
IL-1β: interleukin-1 beta
μg/mL: micrograms per milliliter
TGF-β: transforming growth factor-beta
CCK-8: cell counting kit-8
hDPCs: human dermal papilla cells
ppm: parts per million
MC3T3-E1: mouse preosteoblast cells
L-929: fibroblasts cells
μM: micromolar
NE: nanoemulsion
NL: nanoliposome
NG: nanogold
A549: human lung cancer cells
MRC-5: normal lung fibroblasts cells
LHN: low-molecular-weight nanoemulsion
ACE: angiotensin-converting enzyme
IC_50_: half-maximal inhibitory concentration
REN: renin
RCTs: randomized controlled trials
EPO: European Patent Office
USPTO: United States Patent and Trademark Office
CDDI: Cortellis Drug Discovery Intelligence
PRISMA: preferred reporting items for systematic reviews and meta-analyses
R&D: research and development
NS: not specified
IPC: International Patent Classification
PVA: poly­(vinyl alcohol)
IP: intellectual property
ISO: International Organization for Standardization
INPADOC: International Patent Documentation
UVB: ultraviolet B radiation.
